# Nerves and Communications

**Published:** 1923-11

**Authors:** 


### NERVES AND COMMUNICATIONS.

THERE is as yet little indication that the nerves of the modern man and the modern woman have become attuned to the shocks which the life of to-day inflicts upon them. Those shocks are, indeed, ever increasing. Vast numbers of people now live at the end of some kind of wire—telegraph wire, telephone wire, or that radio which is constructively, though not actually, a wire. Many of them are tied to all three, and when at intervals they escape from them they are plunged into the midst of a seething street traffic, or forced to hang to the end of a strap which they would willingly lay upon the shoulders of its providers—were those shoulders attainable in concrete form. But a Corporation can neither be kicked nor made to *passer par les étrivières,* and so our nerves continue to be frayed cumulatively without opportunity of retaliation. The consequences are, to say the least, uncomfortable, as every doctor practising in a great agglomeration of population knows only too well. Unless our nerves grow blunter—in itself no very healthy change—they will grow more sensitive even under the trials they are at present compelled to undergo.

Can we, however, be sure that these shocks and trials will not be gradually, or, perhaps, rapidly exacerbated ? We have very recently been threatened with a fresh nervous horror in the shape of a new type of " non-stop " tube train—a train, that is, which merely slows down in the stations while a race of finished acrobats swings itself in and out. But it is not given to all of us to excel in acrobatics —strange as it may seem to the devisers of these atrocities, infirm persons, and people of eighty years of age, often travel by train. What is to happen to them ? Will the railway company provide a staff of respectful young men whose business it will be gently to propel inwards portly elderly ladies or to catch them in their arms that they may not break their necks in alighting ? Even tramcars and omnibuses have stopping places—their conductors, in fact, are charged to prevent passengers from alighting while the vehicles are moving. If experiments with these ever-circulators, which are to slow down to two miles an hour in stations, had not actually been made, we should have taken them to be the invention of some humorist anxious to make our flesh creep. But the passion for ceaseless movement appears to be acquiring new strength. A certain number of tube stations in London have discarded lifts and have adopted moving staircases, and we are now told that the escalator is to become almost universal. We are very sorry to hear it. Many people cannot face the moving staircase with equanimity. They are dizzy for hours after they step off, and often suffer from nausea for the rest of the day. The alternative of walking up a couple of hundred steps is not agreeable, especially to those not in their first youth, but they find it less trying than the unceasing escalator.

Not even with the moving staircase and the non-stop train shall we have reached the end of the new trials to our nerves if the " progressives " have their way. They are now talking about moving " side-walks," by which we suppose they mean footpaths—a thirst for progress ought surely to be compatible with respect for the English language. " If," we are loftily told, " we can step off the moving escalator, we shall also be able to alight from the moving side-walk." But we should not always want to step off it—it is conceivable that some of us might wish to look into a shop-window or converse for a minute or two with a friend or acquaintance casually met. Oxford Street and Regent Street have, probably, the most crowded pavements in London—crowded with ladies, two or three deep, gazing at the sumptuary marvels displayed in the shop-windows for their delectation or destruction. Neither they nor the shopkeepers are likely to see the adoption of moving pavements without a struggle. What we need are wider pavements and wider roads, the banishment of certain classes of traffic from narrow thoroughfares, and some reform in shop windows, which might easily be so internally enlarged that most of the *vitrines* are inside. Such windows already exist and their number will, no doubt, increase. Thus we see that this matter of street and railway traffic is partly one of health and partly one of convenience. Health, in the shape of lessened strain upon the nerves, already sorely tried by the conditions of modern life, must come first, and convenience is more likely to be consulted by reasonable deliberation than by breakneck speed. The saving of half a minute is of no importance beside the saving of a few extra heart-beats or of unnecessary fatigue and excitement.

